# An Often Missed Cause of Chronic Pancreatitis: A Case Report on the Rare Presentation of Primary Hyperparathyroidism

**DOI:** 10.1002/ccr3.70950

**Published:** 2025-09-24

**Authors:** Kareem Ibraheem, Amal M. Shawabka, Fatima Manasrah, Osama Hroub, Abdalrahman N. Herbawi, Badawi Eltamimi

**Affiliations:** ^1^ Faculty of Medicine Palestine Polytechnic University Hebron Palestine; ^2^ Gastroenterology Department Al Ahli Hospital Hebron Palestine

**Keywords:** chronic pancreatitis, endoscopic retrograde pancreatography (ERCP), hypercalcemia, pancreatic duct stones, primary hyperparathyroidism

## Abstract

A 30‐year‐old woman with recurrent pancreatitis and nephrolithiasis was diagnosed with chronic calcific pancreatitis linked to primary hyperparathyroidism (PHPT). Following decompression of the pancreas and parathyroidectomy, her symptoms resolved and PTH levels normalized. This case underscores the importance of recognizing PHPT‐induced hypercalcemia as a potential cause of chronic pancreatitis for effective treatment.

## 1 Introduction

Chronic pancreatitis (CP) is characterized by a variety of symptoms and clinical signs related to functional changes, resulting from glandular fibrosis and atrophy associated with both acute and chronic inflammation [1]. Genetically or anatomically predisposed patients are more susceptible to developing CP, with environmental stressors being the primary triggers. While CP is often preceded by acute pancreatitis (AP), it is increasingly being diagnosed in patients without a history of AP or abdominal pain. Disease‐related complications are usually gradual and irreversible [1].

Primary hyperparathyroidism (PHPT), once considered rare, is now recognized as a common disorder affecting mineral metabolism. The recent increase in its incidence is largely due to routine blood calcium testing and parathyroid hormone (PTH) measurements in osteoporosis evaluations. The global epidemiology of PHPT was not a key focus at the Fourth International Workshop on Asymptomatic PHPT [2].

Although rare, hypercalcemia is a recognized cause of both acute and chronic pancreatitis, with PHPT being the primary underlying cause of hypercalcemia‐induced pancreatitis. However, the frequency of pancreatitis among PHPT patients varies. While most studies suggest an association between the two conditions, some have found pancreatitis incidence rates comparable to the general population [3]. These conflicting findings suggest that other disease‐modifying factors may play a critical role in the development of pancreatitis in patients with PHPT. Molecular research indicates that elevated cytosolic calcium levels contribute to the onset of AP [3].

## 2 Case Presentation

### 2.1 Case History/Examination

A 30‐year‐old woman presented to the emergency department with a complaint of severe and continuous epigastric pain for the past 24 h. The pain radiated straight to her back and was not relieved by analgesics. It worsened after meals and improved slightly when she leaned forward. Her symptoms were associated with nausea and non‐projectile vomiting, with vomitus that was neither bile‐stained nor bloody. Notably, the patient had been hospitalized four times in the past four months for AP of unknown cause and three times for nephrolithiasis. She denied smoking or alcohol consumption. The patient is G2P2A0 without any complications during her previous pregnancies.

On physical examination, the patient was afebrile, with a heart rate of 105 beats per minute, blood pressure of 129/85 mmHg, and a respiratory rate of 19 breaths per minute. Her abdomen was non‐distended, and there were no visible abdominal masses or surgical scars. There was no bruising around her umbilicus or flanks. Bowel sounds were hypoactive, and she exhibited marked tenderness upon palpation of the abdomen without rebound tenderness, guarding, or signs of ascites. The rest of her physical examination was unremarkable.

### 2.2 Methods (Differential Diagnosis, Investigations, and Treatment)

Given her history of recurrent pancreatitis and nephrolithiasis, the differential diagnosis included CP, pancreatic duct stones, and hyperparathyroidism. Laboratory investigations revealed no leukocytosis, with a white blood cell count of 8.2 × 10^3^/μL (normal: 3.80–10.40 × 10^3^/μL), hemoglobin levels of 13.2 g/dL (normal: 12.0–16.0 g/dL), and normal renal function with a creatinine level of 0.9 mg/dL (normal: 0.60–1.20 mg/dL). There were slightly elevated lipase levels at 452 U/L (normal: 11–82 U/L) and amylase levels of 570 U/L (normal: 30–110 U/L), while liver transaminases, bilirubin, alkaline phosphatase, and gamma‐glutamyl transferase were within normal limits. Given her history of nephrolithiasis, a PTH test was conducted, revealing elevated levels at 116.7 pg/mL (normal: 15–68.3 pg/mL), suggesting primary hyperparathyroidism (PHPT).

**FIGURE 1:**
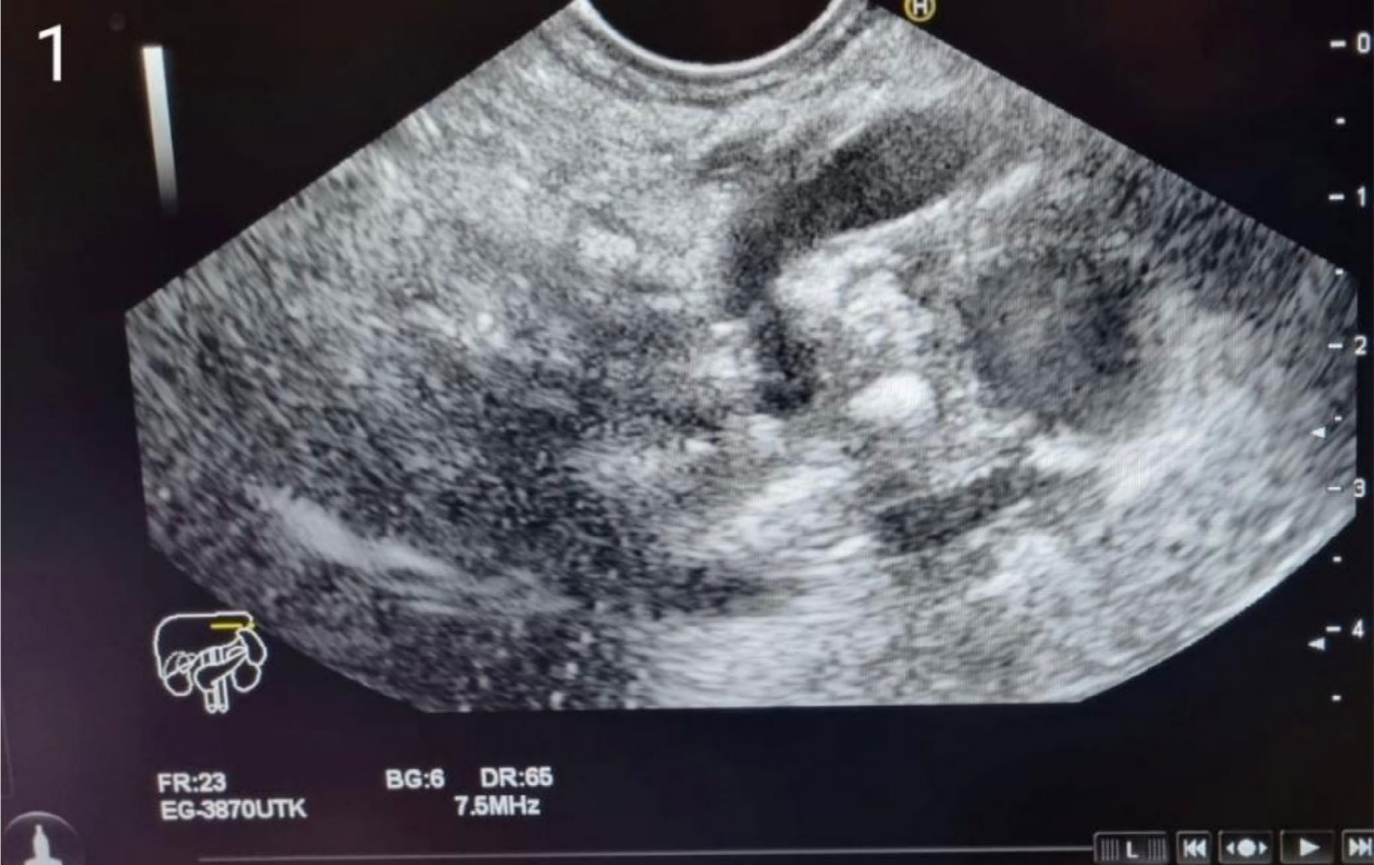
Pancreatic EUS shows mild pancreatic duct dilation, nodular patterns, honeycombing, and extensive parenchymal calcifications.

**FIGURE 2:**
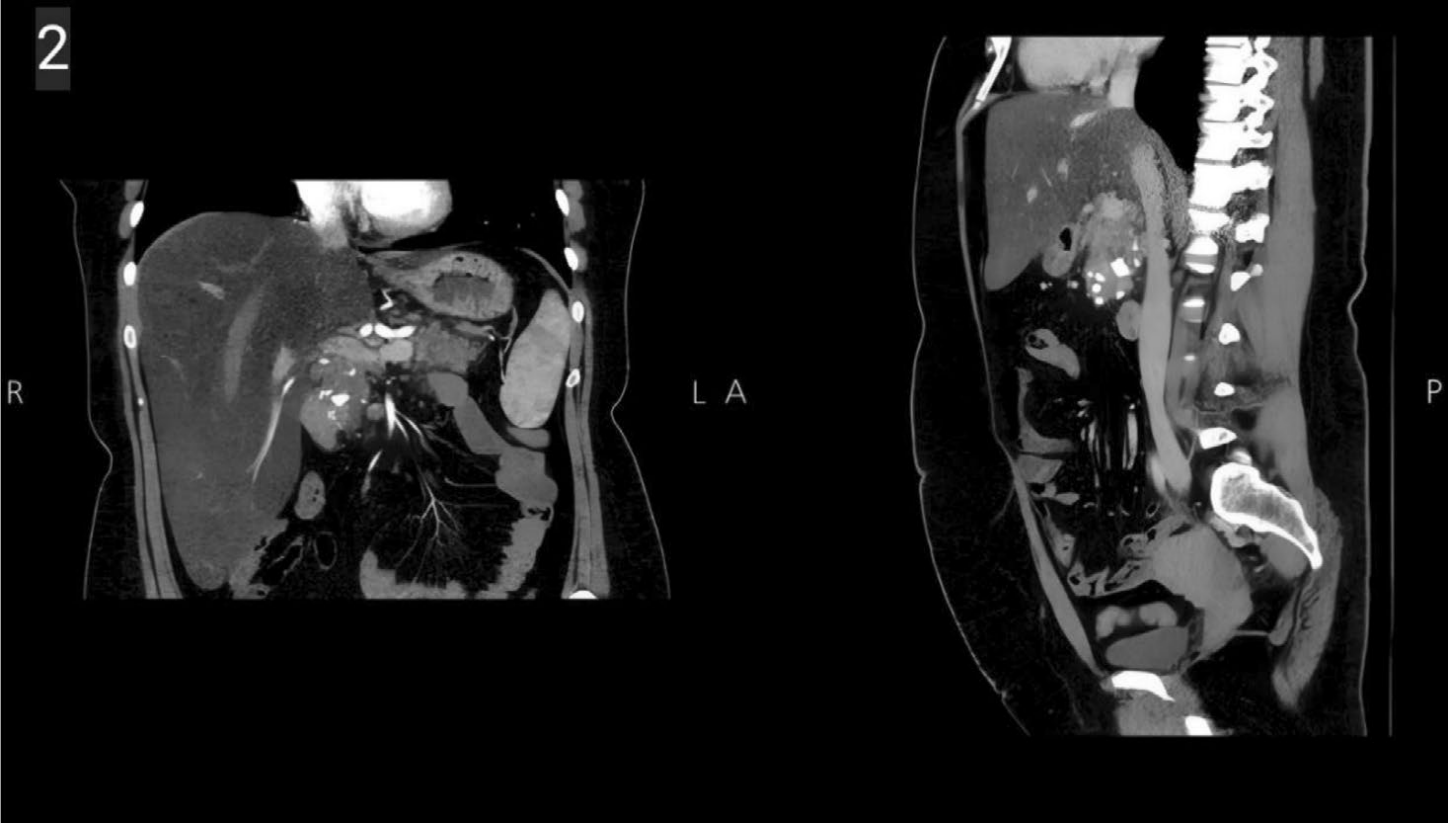
Sagittal and coronal abdominal CT scan images show ductal dilation, multiple intraductal calculi, parenchymal calcifications, and mild peripancreatic fat stranding.

To further evaluate the cause of her recurrent pancreatitis, an endoscopic ultrasound (EUS) revealed a normal‐sized pancreas with significant nodular patterns, honeycombing, and extensive parenchymal calcifications in the head region, measuring 2–7 mm. These findings were consistent with chronic calcific pancreatitis according to the Rosemont classification. Suspected pancreatic duct stones and mild pancreatic duct dilation were noted (Figure 1). A contrast‐enhanced CT scan confirmed the presence of intraductal calculi, parenchymal calcifications, and mild peripancreatic fat stranding, indicating AP. The scan also showed three calcified stones within the pancreatic duct, confirming CP (Figure 2). Cervical ultrasonography identified a heterogeneous hypoechoic mass in the right thyroid bed, measuring 1 × 0.96 × 1.32 cm, suggestive of a parathyroid adenoma, which was confirmed by a neck MRI.

Initial treatment was supportive and conservative, consisting of intravenous fluid resuscitation, bowel rest (NPO), electrolyte repletion, and analgesics for pain relief. The patient's abdominal pain improved gradually, and her lipase levels decreased.

After two weeks of conservative management, the patient underwent endoscopic retrograde pancreatography (ERCP) to relieve the blockage in the pancreatic duct, during which a fully covered metal stent was placed. She then underwent an elective parathyroidectomy for one parathyroid gland to address the hyperparathyroidism, with a biopsy sent to pathology, in which the result showed parathyroid adenoma, confirming the diagnosis of PHPT.

### 2.3 Conclusions and Results (Outcome and Follow‐Up)

Following the ERCP and parathyroidectomy, the patient reported complete resolution of her abdominal pain. Her lipase levels normalized, and she no longer experienced symptoms of pancreatitis. Her PTH levels also returned to normal post‐surgery. In the long term, the patient was advised to make lifestyle changes, including avoiding alcohol and smoking, and following a low‐fat diet to reduce the stress on the pancreas and minimize the risk of future episodes of pancreatitis.

The patient was prescribed analgesics for pain management, pancreatic enzyme supplements to aid digestion, and insulin therapy was considered if diabetes developed due to a pancreatic damage. Nutritional support, including dietary supplements and vitamins, was also recommended to ensure proper nutrition. At follow‐up, the patient remained symptom‐free and in good health.

## 3 Discussion

This case presents a 30‐year‐old female who has had CP as a complication of PHPT due to parathyroid adenoma. The patient also developed nephrolithiasis with recurring renal colic, all of which were linked to hypercalcemia originating from PHPT. These symptoms highlight the intimate relationship between parathyroid imbalance and pancreatitis, and the necessity for considering the rare causes of pancreatitis in the diagnosis of patients with recurrent episodes of pancreatitis or with CP.

### 3.1 Overview of Pancreatitis and Hyperparathyroidism

Pancreatitis is an inflammatory condition that may be acute or chronic in origin. AP is usually caused by gallstones or alcohol intake (in 70%–80%), while hypertriglyceridemia, medications, and infections are less prevalent (10%) and idiopathic in 10% [4]. CP, on the other hand, is characterized by a continuous inflammation that destroys the pancreas over the years due to damage to and loss of exocrine (acinar), endocrine (islet cells), and ductal cells [5]. It is generally associated with alcohol use, genetics, or metabolic abnormalities such as hypercalcemia. Hypercalcemia induced by hyperparathyroidism is responsible for 1.5%–7% of pancreatitis cases [6]. This relationship is uncommon and urges physicians to explore endocrine etiology, particularly in repeated episodes of pancreatitis.

PHPT is most commonly caused by a solitary parathyroid adenoma (80%), four‐gland hyperplasia accounts for 10%–15%, multiple adenomas (5%), while parathyroid cancer is a rare cause of PHPT, representing less than 1% of cases. The reported incidence of PHPT varies significantly, ranging from around 0.4 to 82 cases per 100,000 individuals, likely due to differences in diagnostic criteria, population screening practices, and access to healthcare [7].

PHPT, which is mainly caused by parathyroid adenoma, leads to hypercalcemia due to the overproduction of PTH. Hypercalcemia can manifest through various clinical conditions, including nephrolithiasis, osteoporosis, neuropsychiatric disturbances, and pancreatitis. PHP diagnosis is defined by high blood calcium and abnormally high PTH levels [8], and the location of the adenoma is determined using ultrasound or sestamibi scanning [9]. There is no strict cut‐off value for serum calcium that definitively causes primary hyperparathyroidism (PHPT), as some cases are classified as normocalcemic PHPT, where serum calcium levels remain within the normal range despite elevated PTH [10]. However, there is evidence suggesting a specific threshold in pregnant women with PHPT‐induced acute pancreatitis (AP), where serum calcium levels are typically above 3.5 mmol/L [11].

### 3.2 The Relationship Between Parathyroid Hormone and Pancreatitis

Hyperparathyroidism is connected with pancreatitis largely because of the effect of hypercalcemia on the pancreas. Hypercalcemia causes pancreatitis by making the pancreatic acinar cell produce abnormal, sustained calcium levels through premature pancreatic protease activation. Although most individuals with hyperparathyroidism never suffer from pancreatitis, those with severe hypercalcemia due to hyperparathyroidism are most susceptible. This link has been documented in the literature, but the specific processes via which this correlation occurs are not well known [12].

### 3.3 Literature Review of Previous Cases

There are some previous case reports that documented some cases of the relationship between hyperparathyroidism and pancreatitis, but they remain few. There is a recent case report described by Mehta et al. [13] for a 52‐year‐old woman who has had three episodes of recurrent pancreatitis over a duration of 6 months, who was then diagnosed with PHPT due to parathyroid adenoma. The patient's calcium and PTH levels returned to normal after the parathyroid adenoma was excised, and subsequent pancreatic surgery helped to ease the patient's pancreatitis. This case is comparable to the present patient, as parathyroidectomy and pancreatic decompression with ERCP likewise resulted in the relief of symptoms.

Another case documented by Kumar et al. involved a 30‐year‐old woman with 4 episodes of recurrent pancreatitis and hypercalcemia. The ultimate diagnosis was PHPT caused by a parathyroid adenoma, and the patient had an accidental pituitary adenoma. After parathyroidectomy, the patient had no more pancreatitis, and all the biochemical markers, including PTH and calcium, were within normal range. The presence of parathyroid and pituitary adenomas raised the potential for multiple endocrine neoplasia (MEN) syndrome but was rejected due to not fulfilling the criteria [14]. While the recent case did not come with MEN syndrome and the pituitary adenoma was an accidental discovery, both examples emphasize the importance of hyperparathyroidism in individuals with recurrent pancreatitis and hypercalcemia.

### 3.4 Treatment of Pancreatitis and Hyperparathyroidism

The treatment of CP focuses on symptom relief, preventing complications, and managing the underlying causes. Key treatments include lifestyle changes, such as avoiding alcohol and tobacco, pain management with analgesics or nerve blocks, and enzyme replacement therapy to improve digestion. In advanced stages, surgery such as ERCP may be necessary to eliminate the blockage in the ducts and promote pancreatic outflow [15]. However, in situations where pancreatitis is caused by hyperparathyroidism, surgery is the preferred therapy strategy. Surgery frequently leads to the normalization of blood calcium and PTH levels; therefore, removing the consequences of hypercalcemia such as pancreatitis [16].

In the current case, the patient had a parathyroidectomy to remove the adenoma and subsequently the patient was exposed to decompression of the pancreatic duct using ERCP. These procedures resulted in stability of her health with the remission of CP and avoidance of recurrent bouts of renal stones.

Therefore, it's critical for physicians to identify hyperparathyroidism in patients who arrive with recurring, unexplained pancreatitis by monitoring serum calcium levels.

## 4 Conclusion

This 30‐year‐old woman presented with severe epigastric pain due to recurrent AP, likely caused by chronic calcific pancreatitis linked to hyperparathyroidism. Imaging confirmed pancreatic duct stones and a parathyroid adenoma. Treatment involved conservative management with fluids, NPO, and analgesics, leading to symptom improvement. ERCP was performed for ductal decompression, followed by elective parathyroidectomy. On follow‐up, she was symptom‐free with normal PTH levels. The treatment of CP aims to relieve symptoms, prevent complications, and address underlying causes. For pancreatitis linked to hyperparathyroidism, surgery is the preferred approach, as it normalizes calcium and PTH levels, alleviating pancreatitis. Therefore, it's crucial for physicians to consider hyperparathyroidism in patients with recurrent, unexplained pancreatitis by monitoring serum calcium levels. Long‐term management for this patient includes lifestyle changes, pain control, pancreatic enzyme supplements, and dietary adjustments to prevent further pancreatic damage.

## Author Contributions

**Kareem Ibraheem:** conceptualization, formal analysis, writing – original draft, writing – review and editing. **Amal M. Shawabka:** data curation, methodology, writing – review and editing. **Fatima Manasrah:** formal analysis, investigation, methodology, writing – original draft. **Osama Hroub:** conceptualization, data curation, formal analysis, writing – original draft, writing – review and editing. **Abdalrahman N. Herbawi:** conceptualization, investigation, writing – review and editing. **Badawi Eltamimi:** conceptualization, data curation, formal analysis, investigation, supervision, validation.

## Consent

Written informed consent was obtained from the patient for the publication of this case report.

## Conflicts of Interest

The authors declare no conflicts of interest.

## Data Availability

The data used to support the findings of this study is included in the article.
